# 

**Published:** 1855-01

**Authors:** 


### AMERICAN JOURNAL

OF

### DENTAL SCIENCE.

EDITED BY

CHAPIN A. HARRIS, M. D., D. D. S.

ALFRED A. BLANDY, M. D., D. D. S.

AND

A. SNOWDEN PIGGOT, M. D.

VOLUME V.—NEW SERIES—No. 1.

Surgeon Gen'l Library Washington D.C.
21690

PHILADELPHIA:
LINDSAY & BLAKISTON.
BALTIMORE:—ARMSTRONG & BERRY.
LONDON:—TRUBNER & CO., 12 PATERNOSTER ROW.
1855.

